# Phytochemical Analysis and Evaluation of the Antioxidant, Antiproliferative, Antibacterial, and Antibiofilm Effects of *Globularia alypum* (L.) Leaves

**DOI:** 10.3390/molecules28104019

**Published:** 2023-05-11

**Authors:** Sahar Nouir, Amal Dbeibia, Rim Bouhajeb, Houda Haddad, Amani Khélifa, Lotfi Achour, Mariem Ghardallou, Amira Zaïri

**Affiliations:** 1 Laboratory of Biochemistry, Faculty of Medicine of Sousse, University of Sousse, Sousse 4002, Tunisia; 2 Laboratory of Analysis, Treatment and Valorization of Environmental Pollutants and Products, Faculty of Pharmacy of Monastir, University of Monastir, Monastir 5000, Tunisia; 3 Laboratory of Bioactive Natural Substances and Biotechnology Research, Faculty of Dental Medicine of Monastir, University of Monastir, Monastir 5000, Tunisia; 4 High Institute of Biotechnology of Monastir, University of Monastir, Monastir 5000, Tunisia; 5 Department of Community Medicine, Faculty of Medicine of Sousse, University of Sousse, Sousse 4002, Tunisia

**Keywords:** *Globularia alypum* (L.), phenolic compounds, antioxidant activity, antiproliferative activity, antibacterial activity, antibiofilm activity

## Abstract

*Globularia alypum* L. (GA) is a Mediterranean plant of the *Globulariaceae* family which is widely used in traditional Tunisian medicine. The main goal of this study was to evaluate the phytochemical composition, antioxidant, antibacterial, and antibiofilm activities, and the antiproliferative potential of different extracts of this plant. The identification and the quantification of the different constituents of extracts were determined using gas chromatography–mass spectrometry (GC-MS). The antioxidant activities were evaluated using spectrophotometric methods and chemical tests. The antiproliferative study was based on the use of colorectal cancer SW620 cells, including an antibacterial assessment with the microdilution method and analysis of the antibiofilm effects via the crystal violet assay. All extracts presented several components, mainly sesquiterpenes, hydrocarbon, and oxygenated monoterpenes. The results revealed that the maceration extract had the most important antioxidant effect (IC_50_ = 0.04 and 0.15 mg/mL), followed by the sonication extract (IC_50_ = 0.18 and 0.28 mg/mL). However, the sonication extract demonstrated significant antiproliferative (IC_50_ = 20 µg/mL), antibacterial (MIC = 6.25 mg/mLand MBC > 25 mg/mL), and antibiofilm (35.78% at 25 mg/mL) properties against *S. aureus*. The results achieved confirm the important role of this plant as a source of therapeutic activities.

## 1. Introduction

Due to their curative properties, medicinal plants have been utilized as treatments for various diseases since ancient times [1]. Nowadays, many people are oriented towards “green therapy” and the use of natural compounds from plants that are found to be free from side effects and less toxic than commercial drugs [2]. The beneficial effects of medicinal plants have mainly been attributed to several compounds, such as polyphenols, flavonoids, and phenolic acids [2]. These compounds have a variety of beneficial effects on human health [3]. Previous studies have reported that the consumption of plants and particularly phenolic compounds has been used for the treatment and prevention of a wide spectrum of chronic diseases, including hypertension and hyperglycemia [4]. For this reason, several scientists have conducted extensive studies using different plant extracts to evaluate the antioxidant, antibacterial, anti-inflammatory, and many other medicinal properties of these extracts [5]. *Globularia alypum* L. Extracts may be candidates for such assays. *G. alypum* is a medicinal plant species belonging to the *Globulariaceae* family which grows in the Mediterranean basin. In Tunisia, it is known as “Zriga” and has been used in folk medicine for several diseases, such as kidney disease, cardiovascular disease, constipation, and diabetes [6]. Its leaves have long been used as hypoglycemic, laxative, and purgative agents [6]. According to biological activity studies, *G. alypum* has diverse biological activities, such as antioxidant, antiobesity, antihyperglycemic, antihyperlipidemic, antipyretic, and analgesic properties [6,7]. Numerous studies have reported that *G. alypum* extracts are in secondary metabolites including flavonoids, tannins, and anthocyanins, which explains their numerous benefits and therapeutic properties [6,8]. However, several studies have reported that the extraction processes and techniques influence the nature and amount of secondary metabolites and have noticeable effects on the recovery of phytochemical contents. Thus, the choice of an adequate extraction method and solvent is necessary to obtain multiple secondary metabolites and for the desired activities [9,10]. In this context, the aim of the present study was to conduct a phytochemical analysis of extracts from the leaves of *G. alypum* and to evaluate their antimicrobial, antioxidant, and antiproliferative activities. Moreover, a comparative study of the extraction methods, including soxhlet, sonication, and maceration, was carried out.

## 2. Results and Discussion

### 2.1. Yield of Different Extraction Methods

Various extraction techniques were used to obtain the phytochemicals from different plant parts. The selection of the proper extraction method is crucial for the qualitative and quantitative studies of bioactive compounds from plant materials. The extraction yield is dependent on the solvent, the method of extraction, and the extraction conditions, such as temperature and time. The extraction method must provide the recovery of the targeted compounds and must avoid their chemical modification or alteration [11,12]. Therefore, it is necessary to select a suitable extraction method and solvent based on the sample matrix, chemistry of the bioactive compounds, and scientific expertise [13]. According to Table 1, the sonication method has the most significant yield (64.8%). This extraction technique provides extracts in high yields with minimal changes in the functional properties of the extract [14]. However, the soxhlet technique uses a higher temperature for a longer period of time, which can alter the quality of the phenolic compounds. The study of Khlifi and his colleagues showed that methanol extract obtained using maceration had a lower yield compared to the one reported in our study (42.4 and 48.8%, respectively) [15]. Our results are in line with several studies which reported that sonication provides better yields in a short extraction time compared to the other methods. It has been reported that sonication preserves the nature of compounds that degrade at elevated temperatures [10]. Another study reported that sonication was found to lower the level of energy consumption, quicken the extraction process, and increase the amount of phytochemicals from annatto seeds [16].

### 2.2. GC-MS Analysis

**Figure 1:**
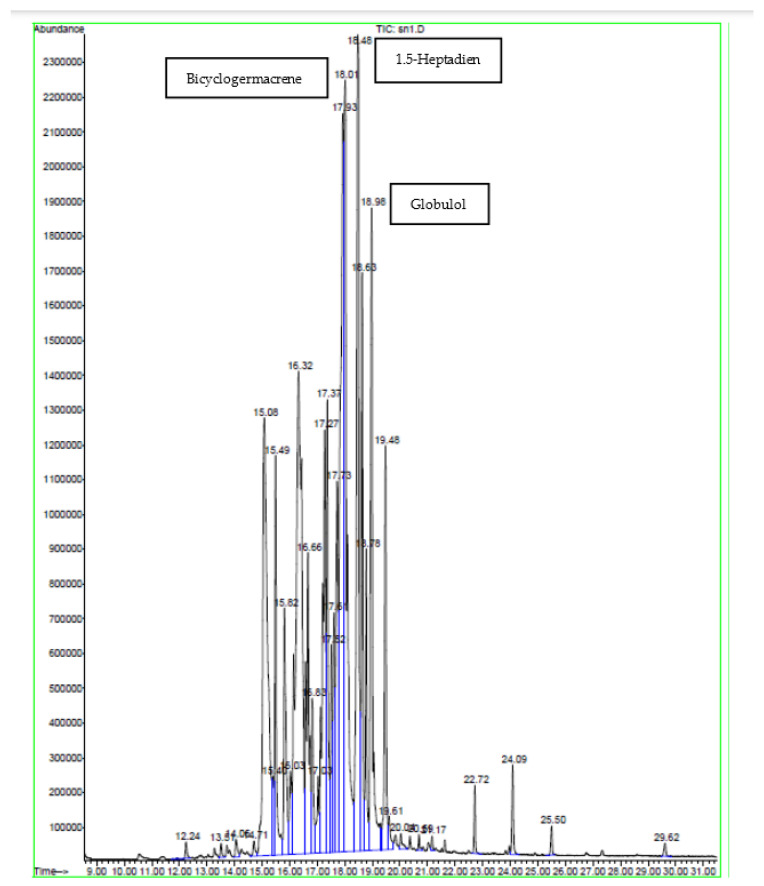
Chromatogram of *G. alypum* leaves obtained via soxhlet extraction.

**Figure 2:**
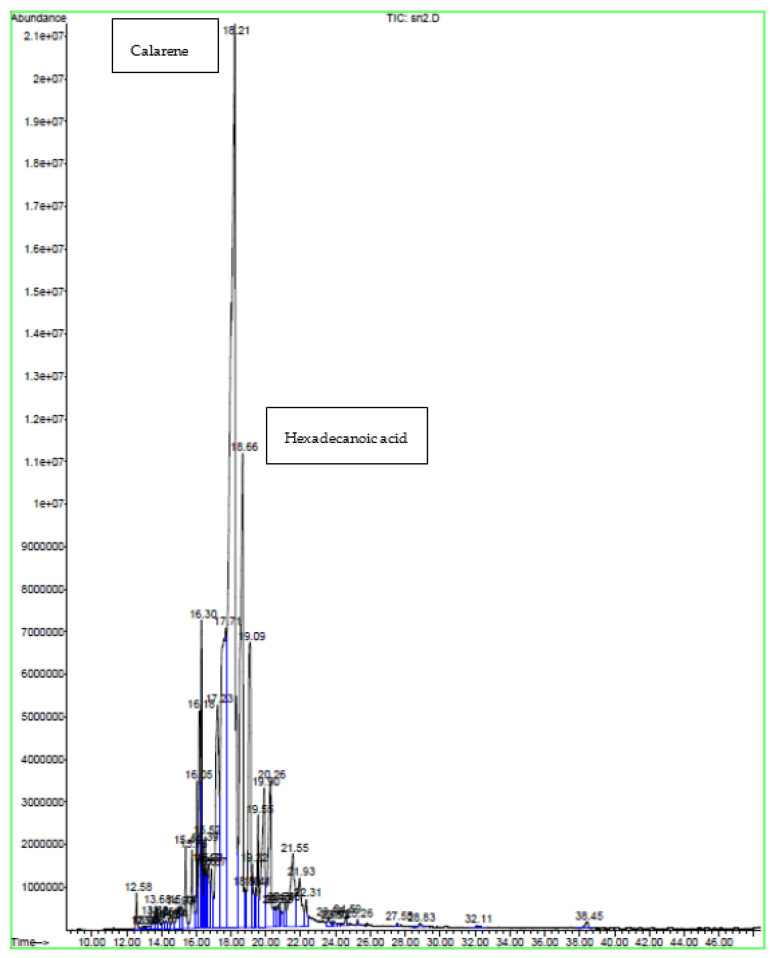
Chromatogram of *G. alypum* leaves obtained via sonication.

**Figure 3:**
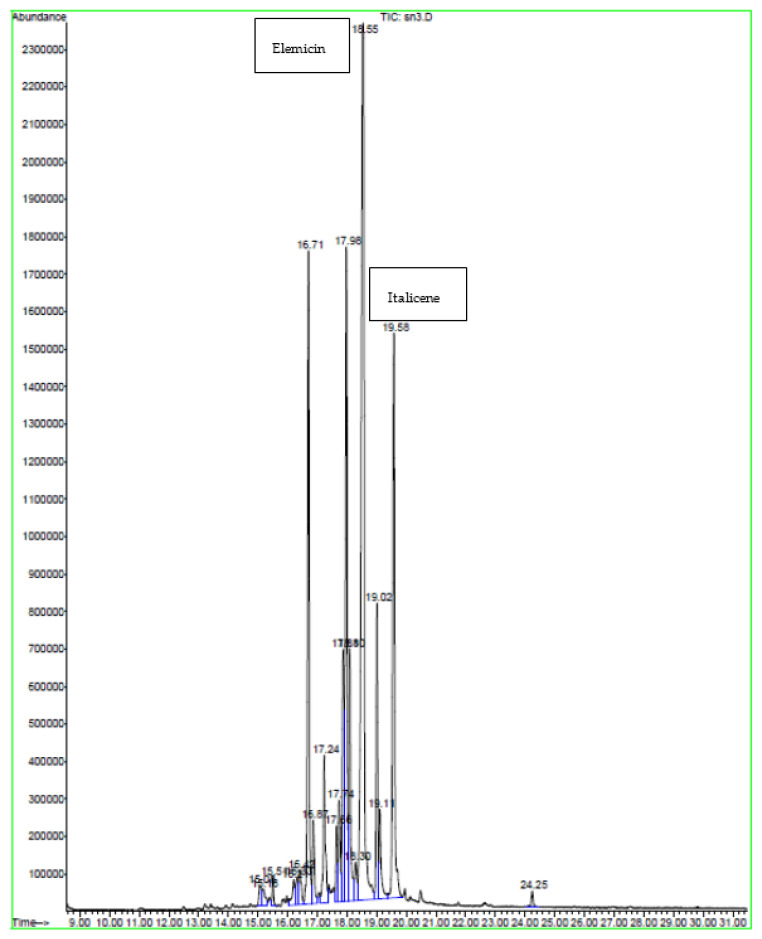
Chromatogram of *G. alypum* leaves obtained via maceration.

GC-MS plays an essential role in the phytochemical analysis and chemotaxonomic studies of medicinal plants containing biologically active components [17]. According to the chromatographic analyses carried out via GC-MS (Table 2, Table 3 and Table 4) and (Figure 1, Figure 2 and Figure 3), various components were identified, including sesquiterpenes, monoterpenes, and other compounds such as organic acids, alcohols, and phenols. Sesquiterpenes were the predominant classes, while hydrocarbons, oxygenated monoterpenes, and other classes were the least characterized. This analysis revealed many different active volatile molecules with different retention times (RT) and different contents (%). Here, 1.5-heptadiene was the most abundant in the extract obtained via soxhlet extraction (content: 13.18%), while clarence and elemicin were the major constituents extracted, respectively, via sonication and maceration (contents: 5.65 and 12.54%). Comparing these three extracts, we determined that calarene was present only in the extract obtained via sonication (content: 5.65%). According to this analysis (Table 2, Table 3 and Table 4), we revealed the presence of a molecule in these three extracts obtained using different techniques and possessing different contents. This molecule was elemicin. It is a phenylpropene known for its antioxidant and antibacterial effects. The extract obtained via soxhlet extraction showed a significant content of β-elemene compared to that obtained via sonication (2.81 and 0.97%, respectively). The study of Al Quahtani and his collaborators, published in 2022, confirmed that β-elemene is a bioactive triterpenoid [18]. Tan and his colleagues demonstrated that β-elemene combined with an IL-23-transfected dendritic vaccine showed inhibitory effects on pancreatic cells and prolonged the survival time, representing an effective antitumor immunotherapeutic method form urine pancreatic cancer [19]. In addition, it was shown that this molecule exerts antitumor effects by decreasing tumor proliferation and metastasis, counting cell apoptosis, and improving cancer sensitivity to chemotherapy [20]. Our result showed a higher content of β-elemene compared with that obtained in the study of Asraoui et al. [21] (β-elemene: 2.81 and 0.03%). Meanwhile, hexadecanoic acid is an abundant molecule, as asserted by Asraoui et al. [21] (hexadecanoic acid: 3.37 and 13.55%, respectively).

### 2.3. Determination of Minerals

Concerning the mineral contents of our extracts, the values of some micro-elements assessed in this study are presented in Table 5. It appears that *G. alypum* has high levels of nitrogen (1.41%) and calcium (1.38%). The amount of minerals in our plant is higher than that observed in other local *G. alypum*, except for calcium. In fact, Khantouche et al. reported that minerals obtained from *G. alypum* from the northeast of Tunisia (Jbel Zaghouan) were 0.04% for P, 0.03 for Na, and 0.48 for P [22]. This difference can be explained by many factors, such as climate, soil composition, and the types of solvents used throughout the extraction protocol. To our knowledge, no other data have been provided concerning the nutrition potential of *G. alypum*. In addition, it is important to mention that the mineral values obtained for our extracts are within the human recommended daily values for minerals, namely, 2000 to 10,000 mg/day for Na; 900–1200 mg/day for Ca; 9–30 mg/day for Fe; 500–1250mg/day for the P; 400–420 mg/day for Mg; 10–20 mg/day for Zn; and 2000 mg/day for K [23].

### 2.4. Antioxidant Activity 

The antioxidant effect of *G. alypum* leaves was evaluated via ABTS and β-carotene assays, and the results were expressed as IC_50_ values (Table 6). As presented in Table 6, the extracts of *G. alypum* showed important antioxidant activities, with significant variability between the extracts and the methods used. The extract obtained via maceration presented the highest antioxidant potential, with values of IC_50_ = 0.04 mg/mL and IC_50_ = 0.15 mg/mL obtained in the ABTS and β-carotene assays, respectively. Meanwhile, the lowest antioxidant activity was observed in the extract obtained via the soxhlet method (IC_50_ = 0.25 and 0.65 mg/mL, respectively). We also noted that the ABTS activity assay was the most sensitive in terms of IC_50_ when comparing the two methods used (Table 6). Phenolic compounds such as flavonoids, phenolic acid, and tannins contribute considerably to the antioxidant activities of medicinal plants [24]. The extract obtained via maceration, which contained the highest level of elemicin, yielded the smallest IC_50_ value.It has been reported that this molecule exhibits several pharmacological effects, including antimicrobial [25] and antioxidant [26] activities. In addition, β-elemene is a sesquiterpene that exerts an antioxidant effect [27]. Furthermore, the study of Trevizan and his collaborators showed that viridiflorol has antioxidant potential [28]. Camphene was also reported to have a strong antioxidant effect [29]. Interestingly, our results suggest that the antioxidant activities of our extracts were significantly higher than those of other local plants. For example, *G. alypum* collected from the northeast of Tunisia (JbelZaghouan) displayed antioxidant activity at IC_50_ = 0.89 and 0.42 mg/mL, as determined via the ABTS and β-carotene assays, respectively [22]. However, values of 0.04 mg/mL and 0.15 mg/mL of our plant were enough to enrich the same effect. The experimental conditions and the methodology used, as well as the use of plants from different geographical regions, could presumably explain the variability observed in the antioxidant activities of *G. alypum*.

### 2.5. Antiproliferative Effect

**Figure 4:**
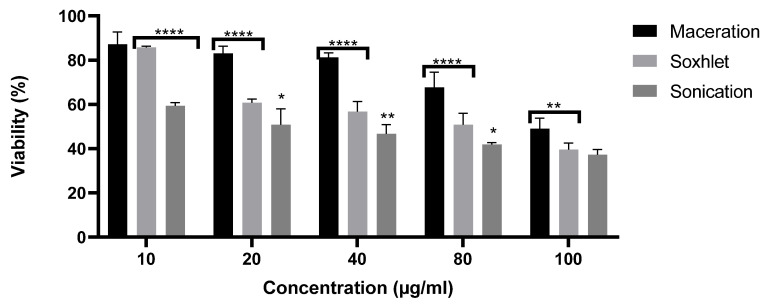
Antiproliferative effect of *G. alypum* leaves obtained via soxhlet extraction. The symbols * and ** indicate a significant (*p* ≤ 0.05) to highly significant **** (*p* ≤ 0.01) difference between different extracts at each concentration. Viability rate (**means ± standard deviations**) of SW620 cells treated with different types of extracts of *G. alypum* leaves. Soxhlet extract (IC_50_= 80 µg/mL), sonication extract (IC_50_ = 20 µg/mL), and maceration extract (IC_50_ = 100 µg/mL). IC_50_: concentration required to reduce SW620 cell viability by 50%.

In the present study, an evaluation of the antiproliferative effects of different extracts from *G*. *alypum* leaves on SW620 was carried out at various concentrations. After 48 h of incubation, the survival rates showed that all the extracts inhibited cell line proliferation in a concentration-dependent manner. The extract obtained via sonication presented the highest anti-proliferative effect (Figure 4), with an IC_50_ = 20 µg/mL. This result can be explained by the richness of this extract of active molecules (Figure 2). Many studies have demonstrated that β-caryophyllene has antioxidant [30], antimicrobial [31], anti-inflammatory [32], and anticancer activities [33]. Comparing our results with those of the study of Friscic and his collaborators, we determined that our extract of *G. alypum* leaves has the most significant antiproliferative effect (IC_50_= 20 and 231.43 µg/mL, respectively) [7].

### 2.6. Evaluation of Antibacterial Activity

In the present research, initial screening was performed to evaluate the antibacterial effects of *G. alypum* leaves on some available bacterial strains (Table 7). All the presented bacteria showed resistance to the extracts, except for *S. aureus*. The extract obtained via sonication showed a significant antibacterial effect on *S. aureus*, with a zone of inhibition of 14.5 mm. The variation in the diameters of the zones of inhibition may be impacted by the microorganisms, the plant, and the antibacterial potential of the bioactive substances of the extract. Several studies have affirmed that the antimicrobial activity is strongly linked to the contents and composition of the extract. Phenolic compounds were also influenced by their diffusion capacity in agar medium [34]. All the tested extracts presented the same MIC and MBC (6.25 and >25 mg/mL, respectively) against *S. aureus* (Table 8). The study of Friscic et al. [7] revealed that the methanolic extract of *G. alypum* leaves obtained via soxhlet extraction had significant antibacterial activity against *S. aureus* in comparison to our results, with MIC= 1.42 mg/mL. According to Lozienne et al. [35], the antibacterial activity depends on several factors, namely, the species of the plant, the preparation of the extract, the solvent used, and the sensitivity of the bacteria. In our study, the antibacterial profiles of the extracts against the tested strains indicated that *S. aureus* is the most sensitive bacterium of all the strains. In other words, the walls of Gram-negative bacteria are more complex than those of Gram-positive bacteria. They act as a diffusional barrier and render cells less sensitive to antimicrobial agents [36]. This difference in sensitivity may also be due to the inhibition of the efflux pump of Gram-positive bacteria [37]. Several works have suggested that the antibacterial activity of flavonoids may be due to the following mechanisms [38]: damage to the cytoplasmic membrane caused by perforation and/or reduced membrane fluidity or the inhibition of nucleic acid synthesis caused by the inhibition of topoisomerase. *P. aeruginosa* has high internal resistance to almost all known antibiotics and antimicrobials, even synthetic drugs, owing to its very restrictive outer membrane barrier, which has been a serious problem worldwide [39]. Regarding our results, the antibacterial activity of the extract obtained via soxhlet extraction could be attributed to globulol, a sesquiterpenoid that was only present only in the extract obtained via soxhlet extraction, with a content equal to 5.47%. In addition, it was reported that globulol has antibacterial activity in the study of Tan and his collaborators in 2008 [40].

### 2.7. Antibiofilm Activity

Our results showed that the antibiofilm effect is dose-dependent (Table 9). After 24 h of culture, the antibiofilm inhibition percentage varied from 35.78% (25 mg/mL) to 16.01% (3.12 mg/mL) for the extract obtained via sonication. The elimination of the biofilm by the extracts may be due to their richness in active molecules such as phenolic compounds [41] and other volatile molecules. Resistance against the various disinfectants is more important in the case of older biofilms than younger ones [42]. This may be related to the fact that the majority of cells in young biofilms are still in the reversible phase of adhesion. In this phase, the bacteria are easily suppressed through the application of minimum forces [43]. Moreover, according to the study of Melo [44], several mechanisms influence bacterial attachment to surfaces, such as the characteristics of the microbial strains, the composition and roughness of the adhesion surface, the availability and concentration of nutrients, surface charge, pH, temperature, electrolyte concentration, and flux of materials, as well as the type of surface.

## 3. Materials and Methods

### 3.1. Plant Material

This study’s use of plant material adhered to the appropriate institutional, national, and international rules and regulations. *G*. *alypum* was taken in the full blooming stage from Tunisia’s Ouardanin area in January 2019 (Table 10). Prof. Fethia Harzallah Skhiri of Tunisia’s High Institute of Biotechnology in Monastir completed the taxonomic identification. The plant voucher specimen was cataloged as Ga 022 in the Herbarium of the Laboratory of Bioresources: Integrative Biology and Valorization (ISBM). Over 7 days, the leaves were washed and dried at room temperature. The material was dried until it reached a constant weight. To optimize the phenolic extraction, the leaves were milled. Three techniques of extraction were used (Table 11). The extracted ethanol was weighed and kept in an amber glass container in a refrigerator (4 °C) after being vaporized using a rotary evaporator. The extract solution was prepared using ethanol absolute for the measurement of active molecules.

### 3.2. The GC-MS Analysis

GC-MS was used to identify the volatile active compounds. The Varian Hp-5890 Gas Chromatograph was used, together with HP5 and Innovax (30 m, 0.25 mm, film thickness 0.25 m) bonded silica capillary columns and FID. The temperatures of the injector and detector were between 240 °C and 280 °C. The volume of nitrogen gas used was 1 mL/min. The oven temperature was set at 50 °C for 3 min and then to 280 °C for 3 min. A total of 0.1 µL of sample diluted in Hexane at a concentration of 1mg/mL was injected, and the integration of peak contents was used to assess the quality and amount of the identified active molecules. To analyse the extracts, we used an HP 5972/A MS set to 70 eV and helium at 20 p.s.i. The identification of volatile molecules was based on a comparison of their retention indices with those of the Wiley Library search routines [45], which were founded on mass spectra that were qualified according to their fit and purity. Kovats index(I) was calculated as I = 100 [n + (Log (t_i_ − t_0_) − Log (t_n_ − t_0_)/Log(t_n+1_ − t_0_) − Log(t_n_ − t_0_)], where n is the carbon number of n-alkane.

The parameters described above in this formula are:

Peak heading: t_i_ is the retention time of compound i in minutes; t_0_ is the air peak void time as the average velocity.

### 3.3. Determination of Minerals

#### 3.3.1. Determination of Potassium and Calcium

The powder materials of both the tested plant species were weighed in a numbered capsule, and then these samples were placed in a muffle furnace at a temperature T1 equal to 220 °C for 2 h and then at a temperature T2 equal to 550 °C for 6 h to ensure the destruction of the chemical bonds and thus obtain free atoms and ions. This step is called calcination. Following this step, concentrated hydrochloric acid was added to each capsule. After heating these samples on a hot plate until the total evaporation of the acid, 5 mL of N/10 hydrochloric acid was added. The next step was filtration. The solutions obtained were filtered into 50 mL volumetric flasks, and distilled water was added to the mark of the dipstick. Finally, the determination of potassium, sodium, and calcium was carried out via flame photometry; therefore, it was necessary to prepare stock solutions and calibration solutions of each element.

#### 3.3.2. Determination of Nitrogen

The determination of nitrogen was carried out according to the method of Martin-Prével et al. [46]. The vegetable powder was mineralized with concentrated sulfuric acid in the presence of a catalyst. The organic nitrogen was transformed into ammonia, and the ammonia was displaced by the soda collected in boric acid. The titration was performed using chloridric acid. The reagents used were concentrated sulfuric acid, 2% boric acid, 40% sodium hydroxide, 15% sodium hydroxide, 0.1 mol/L chloridric acid, and a catalyst. A quantity of 200 mg of the vegetable powder (moisture content between 0 and 10%) was introduced into a flask, avoiding its being deposited on the neck, and then left in contact for 30 min with 5 mL of concentrated sulfuric acid in the presence of 200 mg of catalyst. It was gently heated and then boiled for one hour until a yellow color was obtained. Once the mineralization operation was completed, the tubes were moved one by one into the Vapodest for potential distillation, and the pH was measured.

### 3.4. Antioxidant Activity

#### 3.4.1. ABTS Scavenging Assay

The spectrophotometric analysis of the activity of organic extracts from *G. alypum*, used to trap ABTS + cations, was determined according to the method of El Arem and his collaborators [47]. The ABTS cation radical was generated by mixing equal volumes of a solution of potassium persulfate K_2_S_2_O_8_ and a stock solution of ABTS (39.2 mg), and the combined solution was stored away from light at room temperature for 16 h before use. The solution obtained was diluted with ethanol to obtain an absorbance between 0.7 and 0.8 at 734 nm. A volume of 975 μL of this freshly prepared solution was added to 25 μL of each extract, and the reading was carried out at 734 nm after 20 min for each analysis series. Ascorbic acid was used as a positive control. The antioxidant activity was determined according to the discoloration of the solution and expressed as the percentage inhibition (PI) of the absorbance at the wavelength of 734 nm, at which point the ABTS+• radical presents a characteristic absorption band. Inhibition (%) = [(Abs_control_ − Abs_extract_)/Abs_control_] × 100.

#### 3.4.2. β-Carotene

The oxidation of linoleic acid generated the peroxide radicals which then oxidized the β-carotene, causing the disappearance of its red color. This test was based on an emulsion mixed with 0.5 mg β-carotene, 1mL chloroform, 25 μL linoleic acid, 200 mg Tween 20, and 100 mL distilled water. After that, 2.5 mL of this emulsion was added to 0.5 mL of extract or control BHT (butyl hydroxytoluene) and left to undergo incubation for 2 h at 50 °C in a water bath. The emulsion was added to the sample and measured several times at intervals of 20 min [48] starting with the absorbance at T_0_, which was measured at 490 nm. The percentage of inhibition of peroxidation was calculated according to the following formula: % IP = β-carotene after 2 h/initial β-carotene × 100.

### 3.5. Antiproliferative Effect

An evaluation of the antiproliferative effect was performed using the MTT test for the determination of the cellular metabolic activity with a measure of the SW620 cells extracted from colorectal human cells. These cells were cemented on a plate (MultiScreen^®^filtration plates, 96-well plates) at a density of 5 × 10^3^ in an incubator at 37 °C with 5% CO_2_. The cells were extracted from the medium culture and treated with the extracts (diluted in DMSO) at different concentrations (100; 50; 40; 20; 10 µg/mL). This assay was repeated three times. The treatment with MTT solution (1 mg/mL in culture medium named RPMI 1640 supplemented with 10% fetal bovine serum and 10 µg/mL antibiotic: 5 µg/mL penicillin and 5 µg/mL streptomycin) began after 48h of incubation. The appearance of purple formazancrystals from the yellow tetrazolium salt was an indicator of metabolically active cells. To calculate the percentage of survival of these cells, we used the ratio (OD in the test group/OD in the control group × 100), which was measured using an ELISA reader at 570 nm after 2 h of incubation in an incubator at 37 °C with 5% CO_2_. Three independent experiments were performed.

### 3.6. Antibacterial Activity

Screening was performed using the agar disc diffusion method against the following strains [49]: *Staphylococcus aureus* ATCC 25923, *Salmonella typhimurium* ATCC 14080, *Escherichia coli* ATCC25922, and *Pseudomonas aeruginosa* ATCC 27853. The inoculums of the pathogenic bacteria were adjusted to 0.5 McFarland standard turbidity and then streaked onto Muller–Hinton (MH) agar plates using a sterile cotton mop. Sterile filter discs (diameter 6 mm, Biolife Italy) were placed on the surface of the agar mediums, and 20 µL of each extract diluted in sterile distilled water at 25 mg/mL was dropped onto each disc. Tetracycline (10 mg/mL; 10 μL/disc) was used as a reference antibiotic. After incubation at 37 °C for 24 h, the antibacterial activity was evaluated by measuring the inhibition zone formed around the disc. Each assay was performed in triplicate. The MIC was evaluated [50]. Serial dilutions of the extracts (0.05–25 mg/mL) were applied to plates with 96U-bottomedwells (Nunc, Roskilde, Denmark) together with MH broth and the target bacteria. After incubation at 37 °C for 24 h, the MBC was evaluated by transferring 10 µL showing no bacterial growth after the MIC assay on MH agar from the well. After 24 h of incubation at 37 °C, the bacterial growth was examined, and the MBC was determined as the lowest concentration of the sample having bactericidal activity. All assays were performed in triplicate. Three independent experiments were performed.

### 3.7. Antibiofilm Assay

The antibiofilm assay was determined via the crystal violet assay [50]. The extracts diluted in sterile distilled water at different concentrations (0.05–25 mg/mL) were mixed with pathogenic bacteria suspension (grown in BHI, for 24 h at 37 °C; 10 5 CFU/mL) on plates with 96 U-bottomed wells (Nunc, Roskilde, Denmark) containing brain heart infusion (BHI) (Oxoid) with 2% glucose (*w*/*v*).Wells containing only BHI with 2% glucose and BHI with 2% glucose inoculated with the pathogenic strain served as the negative and positive controls, respectively. After incubation at 37 °C for 24 h, the plates were rinsed 3 times with PBS. Cells in the biofilm were fixed with methanol for 15 min, air-dried, and stained with 1% crystal violet. Biofilm formation was quantified by measuring the absorbance at 595 nm using a microplate reader (GIO. DE VITA E C, Roma, Italy). All assays were performed in triplicate. Three independent experiments were carried out. Inhibition (%) = (OD_control_−OD_Extract_)/OD_control_ × 100.

### 3.8. Statistical Analysis

The results are represented as mean values ± standard deviation. Statistical analyses were based on SPSS version 22. ANOVA and the Student–Newman–Keuls test were used in these analyses. Data were considered statistically different at a *p*-value of 0.05 or less.

## 4. Conclusions

Based on the results of this study, the chemical analysis of *G. alypum* leaves showed the plant’s richness in polyphenols extracted with different methods. In addition, GC-MS analysis revealed the presence of other active molecules. The ethanol extract obtained via maceration had a powerful ability to inhibit oxidation. In addition, the extracts obtained via soxhlet extraction and maceration had important anti-inflammatory effects. However, the extract obtained via sonication demonstrated a significant antiproliferative property against SW620. Because of the richness of *G. alypum* leaves in active molecules, this medicinal plant could be exploited not only for the treatment of diseases related to oxidative stress and colorectal cancer but also for use against bacterial infections. Globally, the sonication method enables the bursting of plant cells by releasing the active molecules through vibration. Comparing these three extraction methods, our results revealed that the extract obtained via sonication presented the highest potential, while the soxhlet method altered the quality of the active molecules due to the long extraction time and the high temperature required. However, the maceration extract, obtained at room temperature, is not the best for evaluating antimicrobial activity.

## Figures and Tables

**Table 1:** Yield percentage using different methods of extraction.

Extraction Method	Yield %
Soxhlet	63.2 ± 0.07 ^b^
Sonication	64.8 ± 0.45 ^a^
Maceration	48.8 ± 0.04 ^c^

Results of the ANOVA test are significantly different at *p* < 0.05, and each data point is represented as the average of three repetitions ± SD of one independent experiment. ^a^: The highest value; ^b^: The medium value; ^c^: the lowest value.

**Table 2:** GC-MS analysis of *G. alypum* leaves obtained via soxhlet extraction.

Peak	Compound	Similarity %	RT	Content %
1	Bicyclogermacrene	95	15.08	9.65
2	β-Elemene	99	15.49	2.81
3	Pentadecane	60	15.81	2.43
4	1.5-Heptadiene	91	16.32	13.18
5	Elemicin	99	16.66	3.75
6	Dendrolasin	90	16.83	1.18
7	Globulol	97	17.28	5.47
8	Viridiflorol	97	17.37	3.57
9	γ-Gurjunene	90	17.52	1.48
10	γ-1-cadinene	70	17.62	1.90
11	2-Pentadecanone	93	18.78	1.84

**Table 3:** GC-MS analysis of *G. alypum* leaves obtained via sonication.

Peak	Compound	Similarity %	RT	Content %
1	β-elemene	91	15.39	0.79
2	β-Caryophyllene	95	15.76	1.10
3	Camphene	92	16.06	1.13
4	Valencene	87	16.18	1.91
5	Zingiberene	90	16.30	3.14
6	Elemicin	98	16.52	0.72
7	Elemol	89	16.88	0.82
8	Calarene	94	17.23	5.65
9	Hexadecanoic acid	99	20.25	3.37
10	Carbonic acid	95	21.55	2.64

**Table 4:** GC-MS analysis of *G. alypum* leaves obtained via maceration.

Peak	Compound	Similarity %	RT	Content %
1	α- Farnesene	76	16.42	0.92
2	Elemicin	99	16.70	12.54
3	Elemol	91	16.87	1.87
4	Spathulenol	99	17.24	3.28
5	Isospathulenol	94	17.66	1.20
6	Italicene	72	17.87	5.09

**Table 5:** Mineral contents of *G. alypum* leaves.

Minerals	N (%)	P (%)	K (%)	Ca (%)	Na (%)	Fe (%)
Content	1.41	0.04	0.61	1.38	0.05	0.017

N: nitrogen; P: phosphorus; K: potassium; Ca: calcium; Na: sodium; Fe: iron.

**Table 6:** Antioxidant activity of *G. alypum* leaves.

IC_50_ (mg/mL)	Ascorbic Acid	Soxhlet	Sonication	Maceration
ABTS scavenging assay	0.06 ± 0.02 ^b^	0.25 ± 0.01 ^d^	0.18 ± 0.00 ^c^	0.04 ± 0.01 ^a^
β-carotene	0.59 ± 0.00 ^c^	0.65 ± 0.05 ^d^	0.28 ± 0.09 ^b^	0.15 ± 0.00 ^a^

Results of the ANOVA test are significantly different at *p* < 0.05, and each data point is represented by the average of three repetitions ± SD of one independent experiment. IC_50_: half maximal inhibitory concentration. Values with different superscripts within the same column are significantly different (*p* < 0.05).

**Table 7:** Evaluation of antibacterial activity of *G. alypum* leaves at 25 mg/mL.

Zone of Inhibition (mm)				
Strains	Tetracycline	Soxhlet	Sonication	Maceration
*E.coli*	29.10 ± 0.00	N	N	N
*P. aeruginosa*	27.20 ± 0.53	N	N	N
*S. typhimurium*	25.01 ± 1.02	N	N	N
*S. aureus*	21.00 ± 1.82 ^a^	11.2 ± 0.30 ^d^	14.50 ± 0.30 ^b^	12.00 ± 0.06 ^c^

Values are means of triplicate determination (n = 3) ± standard deviation. N, no zone of inhibition was found. Values with different superscripts within the same column are significantly different (*p* < 0.05).

**Table 8:** MIC and MBC determination of *G. alypum* leaves.

Strains	*E. Coli*	*P. aeruginosa*	*S. typhimurium*	*S. aureus*
MIC (mg/mL)				
Soxhlet	N	N	N	6.25
Sonication	N	N	N	6.25
Maceration	N	N	N	6.25
MBC (mg/mL)				
Soxhlet	N	N	N	>25
Sonication	N	N	N	>25
Maceration	N	N	N	>25

Minimum Inhibitory Concentration (MIC) and Minimum Bactericidal Concentration (MBC).

**Table 9:** Percentage of biofilm inhibition after 24 h.

Concentration (mg/mL)	25			12.5			6.25			3.12		
Methods	So	S	M	So	S	M	So	S	M	So	S	M
*E.coli*	N	N	N	N	N	N	N	N	N	N	N	N
*S. typhimurium*	N	N	N	N	N	N	N	N	N	N	N	N
*S. aureus*	20.49	35.78	27.19	15.06	28.15	20.01	10.59	20.3	17.10	8.50	16.01	10.24
*P.aeruginosa*	N	N	N	N	N	N	N	N	N	N	N	N

N: no activity. So: Soxhlet. S: sonication. M: maceration.

**Table 10:** Collection site of *G. alypum* L. and its eco-geographical characteristics.

Collection Site	Geographical Location		
	Longitude (E)	Latitude (N)	Altitude (m)
Ouardanin	10°40′35″	35°42′35″	75

**Table 11:** Extraction methods of *G. alypum* leaves.

Extraction Methods	Soxhlet	Sonication	Maceration
Solvent	Ethanol	Ethanol	Ethanol
Temperature (°C)	40	25	25
Time (min)	60	10	240
Plant: solvent proportion	5:100	5:100	5:100
Centrifugation	Not applicable	5 min at 5000 rpm and 4 °C	Not applicable
Equipment	Not applicable	Ultrasound bath (130 kHz)	Not applicable

## Data Availability

Materials, data, and associated protocols are available to readers without undue qualifications regarding material transfer agreements. For data retrieval, please contact (email: zairi_amira@yahoo.fr).
